# Infection clusters can elevate risk of diagnostic target failure for detection of SARS-CoV-2

**DOI:** 10.1371/journal.pone.0264008

**Published:** 2022-02-16

**Authors:** Denise Lopez, Jill Roberts, Marie Bourgeois, Joshua Kootstra, Sharon Minnick, Allison Black, Joshua Mauss, Nick Flores

**Affiliations:** 1 College of Public Health, University of South Florida, Tampa, FL, United States of America; 2 Public Health Branch, County of Tulare, Tulare, CA, United States of America; 3 CZBiohub, San Francisco, CA, United States of America; University of California San Diego, UNITED STATES

## Abstract

The C29197T mutation is one of 4 point mutations known to cause N-gene target failure (NGTF) in the Xpert Xpress SARS-CoV-2 and Xpert Omni SARS-CoV-2 assays from Cepheid (Sunnyvale, CA). We describe a high local prevalence in January of 8.5% (CI 4.9–14.2%) for the C29197T mutation, which was over 3-fold higher than the prevalence estimated statewide in California during the same time frame, 2.5% (CI 2.1–2.8%). Using phylogenetic analysis, we discovered that this increase in prevalence was due, at least in part, to a disproportionately large infection cluster of unknown origin. This study emphasizes the importance of sequencing at the local jurisdictional level and demonstrates the impact that regional variation can have when assessing risk due to point mutations that impact clinical test performance. It also reinforces the need for diligent reporting of abnormal test results by clinical laboratories, especially during Emergency Use Authorization (EUA) periods, as additional information is gathered about the target organism and the performance of EUA-authorized tests over time.

## Introduction

Severe acute respiratory syndrome coronavirus 2 (SARS-CoV-2), which caused a global pandemic of coronavirus disease 2019 (COVID-19), was first detected in Wuhan, China in December, 2019 [1, 2]. Since the start of the pandemic, molecular methods that directly detect the virus in clinical samples have played an important role in the diagnosis and detection of infections to help mitigate spread. The Xpert Xpress SARS-CoV-2, Xpert Xpress DoD, and Xpert Omni SARS-CoV-2 are examples of tests that detect the presence of the SARS-CoV-2 virus based on the chemical detection of its E and N2 gene markers (Cepheid, Sunnyvale, CA). All three of these tests rely on the detection of the N2 gene as one of their two markers for returning a positive result, though failure to detect the N2 gene combined with a detection of the E gene leads to a presumptive positive in the Xpert Xpress SARS-CoV-2 and Xpert Xpress DoD tests, while the Xpert Omni SARS-CoV-2 will return a positive result if the E gene is detected and the N2 gene is not. The utility of these tests and many like them is due, in part, to the high level of specificity achievable with primer and probe design for these types of assays. However, a risk associated with this high level of specificity is susceptibility to false-negatives due to point mutations that may inhibit amplification and/or detection [3–5].

More than one target is recommended for SARS-CoV-2 clinical molecular assays because reliance on a single target can increase the risk of false-negatives [6]. Both the Xpert Xpress SARS-CoV-2 and Xpert Omni SARS-CoV-2 assays use two targets, one within a region coding the nucleocapsid (N) protein and one within a region coding the structural envelope (E) protein, but the exact locations are proprietary [7]. However, already there have been four different point mutations discovered within the N gene that each cause N-gene target failure (NGTF) in these two assays. NGTF is defined here as adverse and abnormal performance of the N-gene target, resulting in either false-negatives or abnormally high cycle threshold (Ct) values compared to the E-gene target (>15 Ct difference). The first mutation of this kind, G29140T, was described by Vanaerschot, Mann [8] in samples from California, USA. Two of these point mutations occur at the same nucleotide position, C29200T first described by Ziegler, Steininger [9], and C29200A described by Hasan, Sundararaju [10]. The fourth, and most recent mutation identified is C29197T [11, 12]. Although there have been cases of target failure of the E-gene in the cobas SARS-CoV-2 test by Roche Diagnostics (Basel, Switzerland), to date there are no documented point mutations that cause failure of the E-gene target in either the Xpert Xpress SARS-CoV-2 or Xpert Omni SARS-CoV-2 assays [13, 14].

We describe a period prevalence rate of the C29197T mutation over 3-fold higher than the statewide prevalence rate for this mutation and that exceeded the 5% rate recommended by the FDA as a trigger for additional caution and assessment of assay performance [15]. This illustrates the importance of local genomic sequencing to fully understand the true risk of diagnostic target failures. In addition, the distribution of the mutation occurred across two different lineages circulating between January and April, 2021. Current methods to monitor genomic characteristics of the SARS-CoV-2 virus center on the proportion of lineages or variants, rather than specific mutations. Failure to track specific mutations can make assessments of risk associated with the prevalence of point mutations that impact clinical assays in a laboratory’s patient population more challenging.

## Methods

### Sample selection and RNA isolation

The university of South Florida Institutional Review Board determined that the proposed activity, STUDY002184, does not constitute research involving human subjects as defined by the US Department of Health and Human Services and US Food and Drug Administration and consent was therefore waived. Study uses residual specimens and data were analyzed anonymously. Residual clinical respiratory swab specimens (Nasal, Nasopharyngeal, and throat) that tested positive by an Emergency Use Authorization (EUA)-approved real-time RT-PCR assay with an average Ct value of ≤32 were selected for sequencing. Samples were left-over from clinical testing performed at local hospitals and the Tulare County Public Health Laboratory. After clinical testing was complete, and before sequencing, positive samples were stored for between 3–30 days at 4°C before being transferred to -80°C. Samples represented residents of the southern San Joaquin Valley of Ca, with most (88.1%) from Tulare County, CA. Samples were extracted using either the Qiagen (Hilden, Germany) QIAmp Viral RNA Mini Kit following manufacturer’s instructions for the manual spin procedure or by the Thermo Fisher Scientific (Waltham, MA) MagMAX Viral/Pathogen Nucleic Acid Isolation Kit on the Thermo Fisher Scientific (Waltham, MA) Kingfisher Flex using the MVP_2Wash_200_Flex.bdz protocol.

### Library preparation and sequencing

Libraries were generated using the ARTIC-NEB: SARS-CoV-2 Library Prep V.4, as described by Mwakibete et al., 2021 [16]. In summary, RNA is reverse transcribed to cDNA and subsequently used to generated ARTIC V3 amplicons. Amplicons are then fragmented and adapter-ligated using the NEBNext Ultra II FS DNA library kit with the following modifications: 1) the volume of water for steps 5.10 and 5.13 were cut in half to 35μl and 32μl respectively, and 2) input Amplicon concentration at step 7 was loaded at 5-50ng instead of 10-100ng. All thermocycling steps were done on the Bio-Rad (Hercules, CA) C1000 touch instrument with the 96 well fast reaction module. Concentrations of the final libraries were determined using the Invitrogen (Waltham, MA) 1x DNA Qubit HS kit on the Invitrogen (Waltham, MA) Qubit Flex instrument. DNA fragment length was determined with the Agilent (Santa Clara, CA) Tapestation using the Agilent (Santa Clara, CA) DNA D1000 ScreenTape kit. Equal amounts (ng) of each sample were then pooled together and diluted to a concentration of 10nM. The Illumina (San Diego, CA) MiniSeq System Denature and Dilute Libraries Guide was then used to prep the final loading concentration of 1.4pM with a 5% phiX spike in. Sequencing was performed on the Illumina (San Diego, CA) MiniSeq platform set up as either a 2X71, 2x100, or 2x146 paired end run with fastq generation, shorter lengths were used to accommodate the custom-made 12bp indices that were used (provided by the Chan Zuckerberg Biohub). Fastq files were then uploaded to the Theiagen (Highlands Ranch, CO) Terra workspace and processed using the Theiagen (Highlands Ranch, CO) Titan_Illumina_PE v 1.4.3 workflow.

### Phylogenetic analyses

To understand the phylogenetic context of viral genomes from the Southern San Joaquin Valley Region, a phylogenetic tree of 2519 SARS-CoV-2 genomes was inferred using the Nextstrain tool suite [17]. Within this dataset, 2269 sequences, including our own, were sampled from California. Another 195 sequences from other areas of the United States were included, and 55 sequences from other countries were included to contextualize variant-of-concern lineages and ensure proper rooting of the tree. The inferential procedure was performed as follows: within Nextstrain Augur sequences were aligned using nextalign v0.1.6, a maximum likelihood phylogenetic tree was constructed with IQTREE v2.0.3 [18], and the molecular clock was used to temporally resolve the tree with TreeTime v0.8.1 [19]. The final trees were exported as JSON files for visualization in Nextstrain Auspice, a Javascript visualization package that enables interactive exploration of the Nextstrain phylogenetic trees. Figures are adapted from Nextstrain visualizations presented in Auspice.

### GISAID data

On May 16, 2021, the GISAID database (gisaid.org) was utilized to determine the prevalence of applicable mutations [20]. Samples that satisfied the following criteria were selected to evaluate prevalence: 1) Identifiable as California in origin; 2) Identifiable to the California county level; 3) Collected between November 1, 2020 –May 16, 2021; 4) Samples did not have ambiguity at the mutation of interest.

## Results

Two upper respiratory samples, both collected the week of March 14, 2021, were forwarded for SARS-CoV-2 PCR testing due to a “presumptive positive” result on the Cepheid Xpert Xpress SARS-CoV-2 assay caused by NGTF. These residual samples were selected for sequencing and both belonged to lineage B.1.1.519. Both shared a single point mutation, C29197T. To investigate the impact of this mutation further, 312 residual upper respiratory SARS-CoV-2 real-time polymerase chain reaction (RT-PCR) samples collected between January 3, 2021 and May 8, 2021 that were sequenced as part of baseline surveillance activities (see S1 Table) were evaluated to determine if they contained the C29197T mutation. Of the 312 samples, 56.1% (n = 175) were indicated by the submitter in the original electronic clinical test order as from an outpatient setting, 2.6% (n = 8) were indicated as from inpatient settings, 1.9% (n = 6) were indicated as from skilled nursing facilities (SNF), 1.6% (n = 5) were indicated as part of healthcare employee screening, 1.0% (n = 3) were coroner specimens, and 36.8% (n = 115) of the samples did not have this data indicated. Of the 312 samples, 59.3% (n = 185) were indicated as being from patients in the age range 25–64, 20.5% (n = 64) in the age range 0–24, and 20.2% (n = 63) in the age range 65 or older. Out of these 312 sequences, 22 (7.1%) of them contained the C29197T mutation. Of note, all 22 C29197T mutants demonstrated NGTF upon secondary testing on the Xpert Xpress SARS-CoV-2 assay (Table 1). Although all 22 C29197T mutants were eventually classified as lineage B.1.1.519, 4 of them were originally classified as B.1.1.222 by an earlier Pango lineage classification (Table 1) [21].

**Table 1:** Clinical Samples showing C29197T mutation and NGTF.

GISAID ID#	Pango Lineage (v2.3.2)	Pango Lineage (v2.4.2)	Mutation	E	N2	SPC
hCoV-19/USA/CA-TCPHL-020221-01/2021	B.1.1.222	B.1.1.519	C29197T, G29227T	16.7	0.0	28.4
hCoV-19/USA/CA-TCPHL-030121-01/2021	B.1.1.222	B.1.1.519	C29197T	21.1	0.0	28.6
hCoV-19/USA/CA-TCPHL-030121-12/2021	B.1.1.222	B.1.1.519	C29197T, G29227T, C29167T	26.8	0.0	29.1
hCoV-19/USA/CA-TCPHL-030121-18/2021	B.1.1.222	B.1.1.519	C29197T	18.7	0.0	28.4
hCoV-19/USA/CA-TCPHL-031521-01/2021	B.1.1.519	B.1.1.519	C29197T	28.3	43.7	28.4
hCoV-19/USA/CA-TCPHL-032221-17/2021	B.1.1.519	B.1.1.519	C29197T	21.8	0.0	28.8
hCoV-19/USA/CA-TCPHL-032221-25/2021	B.1.1.519	B.1.1.519	C29197T	15.8	0.0	28.5
hCoV-19/USA/CA-TCPHL-032921-04/2021	B.1.1.519	B.1.1.519	C29197T	16.1	0.0	28.2
hCoV-19/USA/CA-TCPHL-041221-21/2021	B.1.1.519	B.1.1.519	C29197T	13.6	0.0	27.9
hCoV-19/USA/CA-TCPHL-041221-22/2021	B.1.1.519	B.1.1.519	C29197T	27.0	43.7	28.1
hCoV-19/USA/CA-TCPHL-041921-37/2021	B.1.1.519	B.1.1.519	C29197T	14.4	0.0	28.6
hCoV-19/USA/CA-TCPHL-042621-02/2021	B.1.1.519	B.1.1.519	C29197T	19.1	0.0	27.9
hCoV-19/USA/CA-TCPHL-042621-06/2021	B.1.1.519	B.1.1.519	C29197T	22.9	44.1	28.7
hCoV-19/USA/CA-TCPHL-042621-07/2021	B.1.1.519	B.1.1.519	C29197T	19.4	0.0	28.4
hCoV-19/USA/CA-TCPHL-042621-30/2021	B.1.1.519	B.1.1.519	C29197T	18.6	0.0	28.1
hCoV-19/USA/CA-TCPHL-042621-34/2021	B.1.1.519	B.1.1.519	C29197T	17.3	0.0	28.4
hCoV-19/USA/CA-TCPHL-042621-37/2021	B.1.1.519	B.1.1.519	C29197T, G29227T	15.2	0.0	28.1
hCoV-19/USA/CA-TCPHL-050321-04/2021	B.1.1.519	B.1.1.519	C29197T	23.5	41.6	28.9
hCoV-19/USA/CA-TCPHL-050321-09/2021	B.1.1.519	B.1.1.519	C29197T	15.5	0.0	28.2
hCoV-19/USA/CA-TCPHL-050321-11/2021	B.1.1.519	B.1.1.519	C29197T	20.7	0.0	28.5
hCoV-19/USA/CA-TCPHL-050321-18/2021	B.1.1.519	B.1.1.519	C29197T	QNS	QNS	QNS
hCoV-19/USA/CA-TCPHL-051021-22/2021	B.1.1.519	B.1.1.519	C29197T, G29227T	QNS	QNS	QNS

The 22 C29197T mutants with February 2021 and April 2021 Pango lineage assignments, Xpert Xpress SARS-CoV-2 Ct values, and any additional N-gene mutations within the CDC EUA PCR N-gene target regions. E and N2 are gene targets on the Xpert Xpress SARS-CoV-2 while SPC stands for Sample Processing Control and verifies correct sample processing—the SPC in positive SARS-CoV-2 results may be negative or positive, while they should be positive in a negative sample. Samples listed as QNS (Quantity Not Sufficient) were unable to be screened for N-gene target failure. * Indicates a sample from same household.

In order to refine our estimate of the proportion of C29197T mutants that were circulating among the community, household clusters were identified and eliminated. Out of the 312 samples sequenced for baseline surveillance, there were 26 home addresses and 2 skilled nursing facility addresses that each had more than one sequenced genome collected within a 2-week span. A sample from each household that had the highest quality genome was selected as the representative sample and duplicate samples were removed from the count. Once this was complete, 284 baseline surveillance samples were left, better representing non-household community transmission of SARS-CoV-2. In addition, there was 1 C29197T mutant that shared an address with another and therefore removed from the prevalence count (Table 1). The resulting proportion was very similar and slightly higher, with 21 samples with the C29197T mutation circulating in the community out of the 284 remaining samples (7.4%). Strikingly, the period prevalence for the 142 samples that were collected in January, 8.5% (CI 4.9–14.2%), was particularly high compared to the state rate in California, 2.5% (CI 2.1–2.8%). As a result, phylogenetic analysis was performed to further evaluate the C29197T mutants.

**Fig 1:**
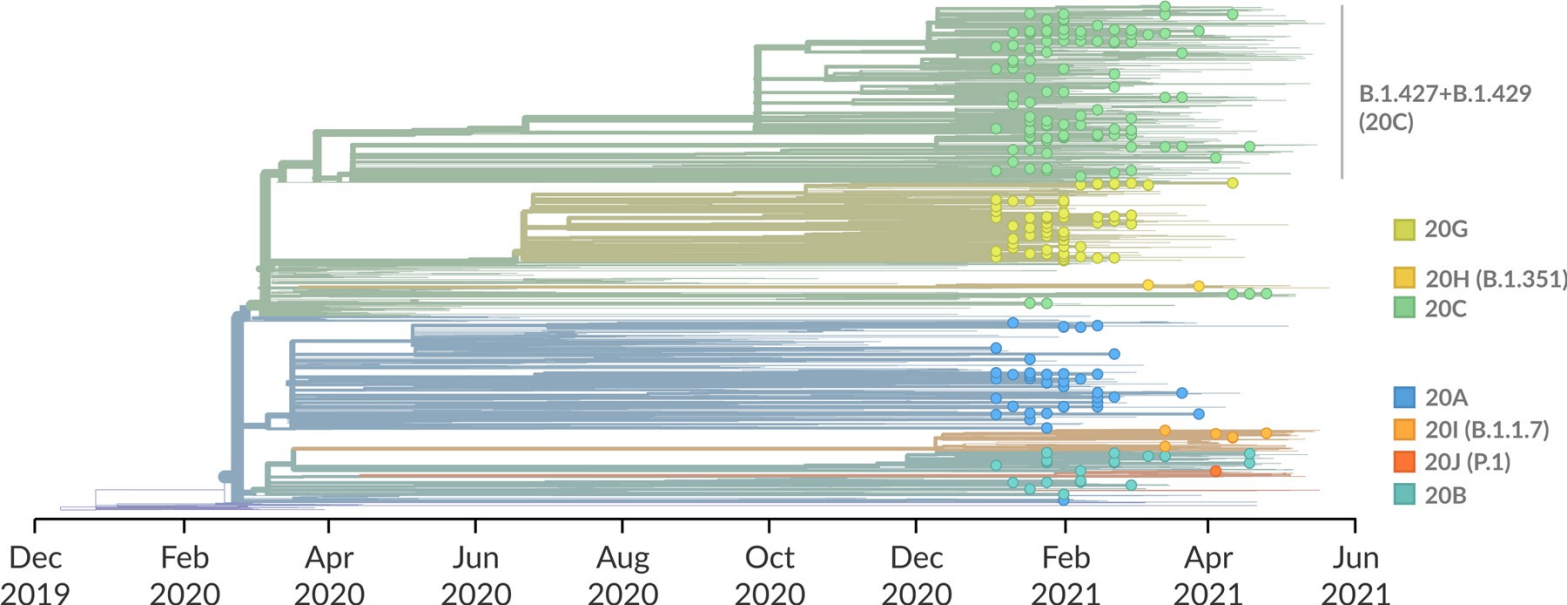
Temporally-resolved phylogenetic tree of 2519 SARS-CoV-2 whole genomes colored by Nextstrain clade designation. The genome sequences generated as part of this study are indicated with circles at the tips. Background sequences are indicated solely by their branch length. Nextrain clade designations, and their counterpart Pango lineages for key clades, are shown to the right of the tree.

Phylogenetic analysis of 275 of the 284 sequenced viral genomes collected between January 3, 2021 and May 8, 2021 from Tulare County indicate that they are a good representation of the broad diversity of SARS-CoV-2 across California (Fig 1). Most of the sequenced viruses clustered in Nextstrain clade 20C (120 viruses, 44%), followed by 20G (66 viruses, 24%), 20A (45 viruses, 16%), and 20B (31 viruses, 11%). Of the 120 clade C viruses, 113 (94%) clustered with viruses from Pango lineage B.1.427/B.1.429 viruses, a variant-of-concern lineage that is frequent within California. Additional variants-of-concern included 9 of the B.1.1.7 lineage, 2 of the B.1.351 lineage, and 1 from the P.1 lineage.

**Fig 2:**
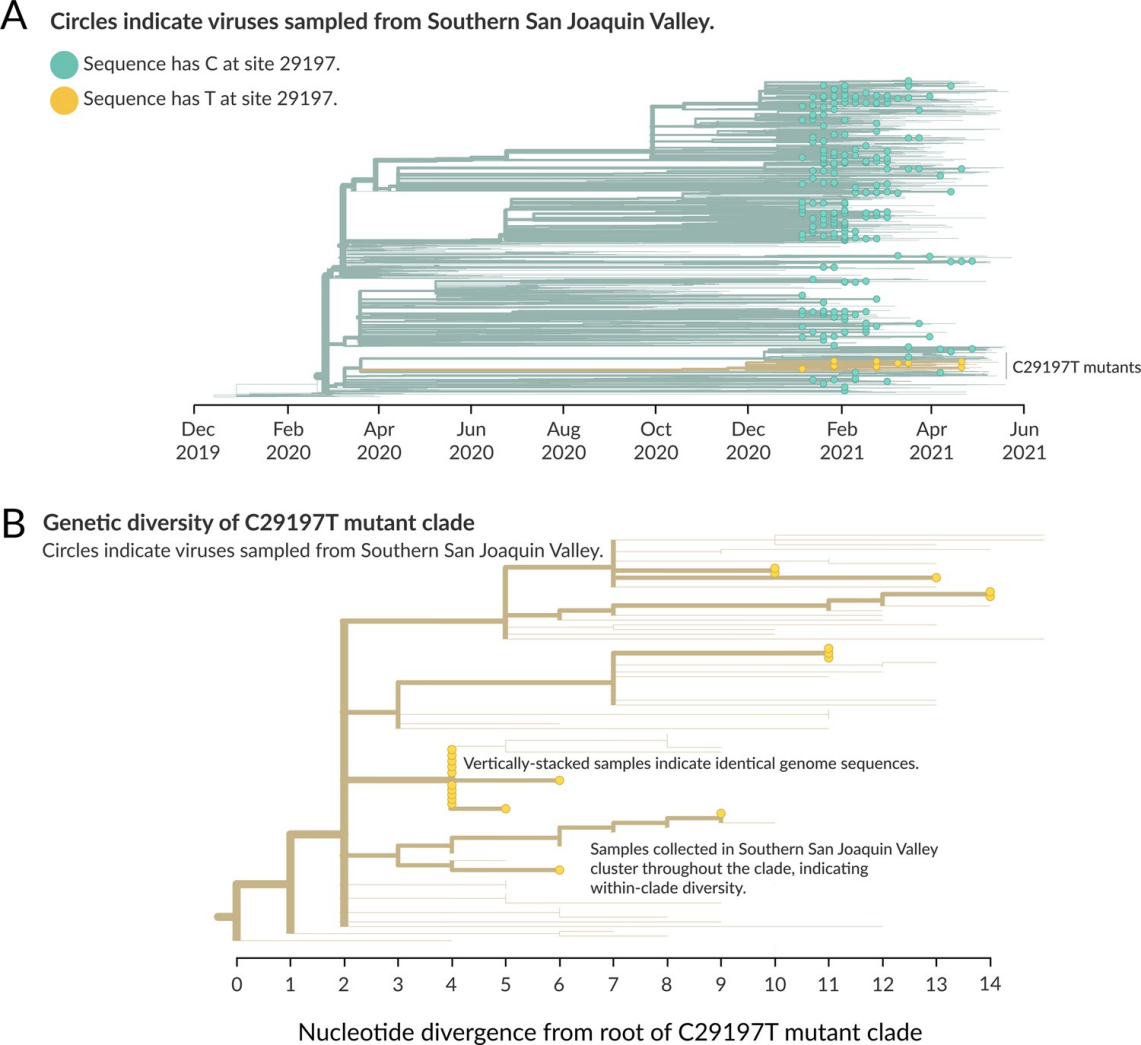
Timing and diversity of 29197T-variant circulation in Southern San Joaquin Valley. (A) Phylogenetic tree of 2519 SARS-CoV-2 genomes colored by the nucleotide found at position 29197 in the genome, indexed per the Wuhan-Hu-1 reference strain (NCBI accession number MN908947). Nucleotide C at position 29197 denotes the wild-type, colored in teal. Nucleotide T at position 29197 denotes the mutation of interest, also referred to as C29197T mutants, highlighted in yellow. As in Fig 1, genomes generated as part of this study have leaves indicated with circles, while background sequences are shown only with branches. (B) Genetic divergence of the C29197T clade, which was made up of both B.1.1.222 variants and B.1.1.519 variants in February before being resolved in April as containing exclusively variants classified as B.1.1.519. Background sequences are indicated only by their branches, while sequences from Southern San Joaquin Valley are indicated with circles at the leaves, and wider branches.

For the most part, the 22 C29197T mutants are relatively diverse and vary genetically in multiple ways from the root of the clade (Fig 2B). Most of these apparent introductions resulted in minimal sequenced transmission, with between one and three sequenced viruses resulting from an introduction. However, one introduction event appears to have been associated with significantly more transmission, leading to 9 infections with identical genome sequences, and a further two infections that appear descended from this introduction event (Fig 2B). A public health investigation showed that all 9 samples were from different households, though an epidemiologic link among the patients within this infection cluster could not be identified.

## Discussion

Local period prevalence of the C29197T mutation for January, 2021, estimated using local baseline surveillance sampling, was over 3-fold higher (8.5%) than the statewide prevalence (2.5%) during the same time frame, estimated using GISAID data. The 95% confidence interval calculated using the local sequencing data exceeded the entire 95% confidence interval of statewide data from GISAID, further indicating the local sequencing efforts captured a significantly elevated prevalence. This high period prevalence well exceeded the 5% prevalence rate set by the United States Food and Drug Administration (FDA) as a threshold to trigger consideration for additional caution and closer assessment of assay performance. This elevated local prevalence rate of the C29197T mutation can be attributed at least in part to a disproportionately large infection cluster of unknown origin of 9 individuals from separate households. Even after controlling for household clusters, only 1 of the original 22 C29197T mutants was from the same household as another, indicative of significant community spread for this mutation. Additionally, the relative diversity of the C29197 mutants observed in this study suggests that multiple introductions of SARS-CoV-2 with the 29197T substitution occurred independently, with multiple separate mutations from the root of the clade associated with this point mutations (Fig 2B). This study demonstrates that local transmission dynamics can significantly impact the prevalence rate of mutations of concern, especially when both transmission rates and viral diversity are high.

Another factor to consider when assessing risk of point mutations such as the C29197T mutation is that 4 of the 22 C29197T mutants were originally classified as variant B.1.1.222 when run on Pango lineage version 2.3.2 in February and as late as March of 2021. This means that a proportion of the B.1.1.222 variants circulating in the state and nationally may have harbored the C29197T mutation as under the previous classification it was not consistent whether B.1.1.222 variants displayed the C29197T mutation or not. When all 22 samples were reprocessed using Pango lineage version 2.4.2 in May 2021, the 4 samples with the C29197T mutation that were originally classified as B.1.1.222 were reclassified as B.1.1.519 and the remaining 18 samples remained classified as B.1.1.519. This is important to note because the main method of monitoring trends using genomic sequencing technology is by using lineage designations, also known as variants. The independent introduction of the C29197T mutation amongst two different, circulating variants would have made it more difficult to identify trends associated with this particular point mutation using conventional variant tracking methods. In fact, national data demonstrated a broad range of proportions of both B.1.1.222 and B.1.1.519 during the months of January–April that likely reflected highly variable local prevalence rates of the C29197T mutation in different regions of the U.S. [22]. This has implications even broader than point mutations that can impact the performance of clinical assays and extends to all mutations of concern.

Pertaining to mutations that can impact the performance of a clinical assay, the overall potential impact on a particular assay must examine the combined prevalence of all point mutations that can impact that assay. In the case of the Xpert Xpress SARS-CoV-2 and Xpert Omni SARS-CoV-2 assays, there are three additional point mutations that have been demonstrated to cause NGTF: G29140T, C29200A, and C29200T. Any one of the 4 documented point mutations can alone cause NGTF in the GeneXpert assay, forcing the assay to rely on a single target for accurate detection. For a region to fully assess the risk of NGTF more accurately it would need to consider the prevalence of all 4 of these mutations. This further demonstrates the challenges of effectively monitoring for mutations of concern.

Another element to consider when assessing potential impact of target failure on assay performance is the sensitivity of the affected target, compared to other targets in the assay. Target failure in the most sensitive assay target would reduce the overall sensitivity of the assay. Sensitivity of an assay target can depend on the quantity of the target viral transcript and specific assay chemistries. The N gene transcript has been quantified as the second most abundant SARS-CoV-2 transcript, after ORF1ab, in nasopharyngeal swabs [23]. In the Cepheid assays, the N2 target demonstrated a lower limit of detection when compared by the manufacturer to the E gene target, as described in the Emergency Use Authorization Instructions For Use, Revision F (January 2021). Failure of the more sensitive of two targets, as described here, increases the likelihood of false-negatives, especially very early in an infection when Ct values can be high.

To minimize the risk of false-negatives, assays that target viruses with high rates of transmission and/or mutation should be designed to be as robust as possible. For example, when designing primers for RNA viruses that have the potential to evolve rapidly, third codon positions should be avoided as primer targets [24]. The third codon position is often more forgiving of mutations and has an elevated risk of accumulating them to a degree detrimental to optimal primer function. In particular, a perfect match at the 3’ end for both primers in a set is critical to rimer function to allow for nucleotide extension by the DNA polymerase [25]. A mismatched site could have a disproportionate impact on primer function so these sites should be designed with care. In addition, it is already best practice to have at least two molecular targets in case of failure in one, especially for a virus with high genetic variability [26]. Until systems to monitor mutations that can impact the performance of clinical assays improve, we recommend three molecular targets be considered by manufacturers, especially when both transmission rate and viral diversity is high. We also recommend that it become best practice for clinical laboratories to ensure that samples that produce abnormal signals in molecular clinical assays be selected for targeted sequencing surveillance. Results from samples that produce similar abnormal results in the same clinical assay should be evaluated together to determine if there is a mutation or set of mutations that may be impacting clinical performance. To help try and address this issue, researchers such as Khan and Cheung [27] developed publicly-available software that any laboratory can use to monitor for mutations that have the potential to impact assay performance. Our analysis of the C29197T mutation demonstrates that timely genomic sequencing information that is available at the local level is crucial to providing actionable information concerning mutations of public health concern.

## Supporting information

S1 Table GISAID identification numbers for the 312 genomes from the southern San Joaquin Valley of California. (DOCX)
