# Supplementary material for: Infection clusters can elevate risk of diagnostic target failure for detection of SARS-CoV-2
Source: PLoS One. 2022 Feb 16;17(2):e0264008. doi: 10.1371/journal.pone.0264008 (PMC8849547; doi:10.1371/journal.pone.0264008)
Supplement: S1 Table — (DOCX) [file pone.0264008.s001.docx]

**S1 Table. GISAID identification numbers for the 312 genomes from the southern San Joaquin Valley of California.**

| hCoV-19/USA/CA-TCPHL-040521-30/2021 | hCoV-19/USA/CA-TCPHL-032221-12/2021 |
| --- | --- |
| hCoV-19/USA/CA-TCPHL-040521-31/2021 | hCoV-19/USA/CA-TCPHL-032221-13/2021 |
| hCoV-19/USA/CA-TCPHL-041221-05/2021 | hCoV-19/USA/CA-TCPHL-032221-14/2021 |
| hCoV-19/USA/CA-TCPHL-041221-06/2021 | hCoV-19/USA/CA-TCPHL-032221-15/2021 |
| hCoV-19/USA/CA-TCPHL-041221-07/2021 | hCoV-19/USA/CA-TCPHL-032221-16/2021 |
| hCoV-19/USA/CA-TCPHL-041221-08/2021 | hCoV-19/USA/CA-TCPHL-032221-26/2021 |
| hCoV-19/USA/CA-TCPHL-041221-09/2021 | hCoV-19/USA/CA-TCPHL-032221-27/2021 |
| hCoV-19/USA/CA-TCPHL-041221-10/2021 | hCoV-19/USA/CA-TCPHL-032221-28/2021 |
| hCoV-19/USA/CA-TCPHL-041221-11/2021 | hCoV-19/USA/CA-TCPHL-032221-29/2021 |
| hCoV-19/USA/CA-TCPHL-041221-12/2021 | hCoV-19/USA/CA-TCPHL-041921-21/2021 |
| hCoV-19/USA/CA-TCPHL-041221-13/2021 | hCoV-19/USA/CA-TCPHL-041921-22/2021 |
| hCoV-19/USA/CA-TCPHL-041221-14/2021 | hCoV-19/USA/CA-TCPHL-041921-23/2021 |
| hCoV-19/USA/CA-TCPHL-041221-15/2021 | hCoV-19/USA/CA-TCPHL-041921-24/2021 |
| hCoV-19/USA/CA-TCPHL-041221-16/2021 | hCoV-19/USA/CA-TCPHL-041921-25/2021 |
| hCoV-19/USA/CA-TCPHL-041221-17/2021 | hCoV-19/USA/CA-TCPHL-041921-26/2021 |
| hCoV-19/USA/CA-TCPHL-041221-18/2021 | hCoV-19/USA/CA-TCPHL-041921-27/2021 |
| hCoV-19/USA/CA-TCPHL-041921-36/2021 | hCoV-19/USA/CA-TCPHL-041921-29/2021 |
| hCoV-19/USA/CA-TCPHL-041921-37/2021 | hCoV-19/USA/CA-TCPHL-041921-31/2021 |
| hCoV-19/USA/CA-TCPHL-041921-38/2021 | hCoV-19/USA/CA-TCPHL-041921-32/2021 |
| hCoV-19/USA/CA-TCPHL-041921-39/2021 | hCoV-19/USA/CA-TCPHL-041921-34/2021 |
| hCoV-19/USA/CA-TCPHL-040521-09/2021 | hCoV-19/USA/CA-TCPHL-042621-09/2021 |
| hCoV-19/USA/CA-TCPHL-040521-10/2021 | hCoV-19/USA/CA-TCPHL-042621-10/2021 |
| hCoV-19/USA/CA-TCPHL-040521-11/2021 | hCoV-19/USA/CA-TCPHL-042621-11/2021 |
| hCoV-19/USA/CA-TCPHL-040521-12/2021 | hCoV-19/USA/CA-TCPHL-042621-12/2021 |
| hCoV-19/USA/CA-TCPHL-040521-13/2021 | hCoV-19/USA/CA-TCPHL-042621-13/2021 |
| hCoV-19/USA/CA-TCPHL-040521-14/2021 | hCoV-19/USA/CA-TCPHL-042621-18/2021 |
| hCoV-19/USA/CA-TCPHL-040521-15/2021 | hCoV-19/USA/CA-TCPHL-042621-19/2021 |
| hCoV-19/USA/CA-TCPHL-040521-16/2021 | hCoV-19/USA/CA-TCPHL-042621-20/2021 |
| hCoV-19/USA/CA-TCPHL-040521-17/2021 | hCoV-19/USA/CA-TCPHL-042621-21/2021 |
| hCoV-19/USA/CA-TCPHL-040521-18/2021 | hCoV-19/USA/CA-TCPHL-042621-22/2021 |
| hCoV-19/USA/CA-TCPHL-040521-19/2021 | hCoV-19/USA/CA-TCPHL-042621-23/2021 |
| hCoV-19/USA/CA-TCPHL-040521-20/2021 | hCoV-19/USA/CA-TCPHL-042621-24/2021 |
| hCoV-19/USA/CA-TCPHL-040521-21/2021 | hCoV-19/USA/CA-TCPHL-042621-26/2021 |
| hCoV-19/USA/CA-TCPHL-040521-22/2021 | hCoV-19/USA/CA-TCPHL-042621-27/2021 |
| hCoV-19/USA/CA-TCPHL-040521-23/2021 | hCoV-19/USA/CA-TCPHL-022221-01/2021 |
| hCoV-19/USA/CA-TCPHL-040521-24/2021 | hCoV-19/USA/CA-TCPHL-022221-02/2021 |
| hCoV-19/USA/CA-TCPHL-040521-25/2021 | hCoV-19/USA/CA-TCPHL-022221-03/2021 |
| hCoV-19/USA/CA-TCPHL-040521-26/2021 | hCoV-19/USA/CA-TCPHL-022221-04/2021 |
| hCoV-19/USA/CA-TCPHL-040521-27/2021 | hCoV-19/USA/CA-TCPHL-031521-11/2021 |
| hCoV-19/USA/CA-TCPHL-040521-28/2021 | hCoV-19/USA/CA-TCPHL-031521-12/2021 |
| hCoV-19/USA/CA-TCPHL-040521-29/2021 | hCoV-19/USA/CA-TCPHL-031521-13/2021 |
| hCoV-19/USA/CA-TCPHL-020221-02/2021 | hCoV-19/USA/CA-TCPHL-031521-14/2021 |
| hCoV-19/USA/CA-TCPHL-020221-03/2021 | hCoV-19/USA/CA-TCPHL-041921-09/2021 |
| hCoV-19/USA/CA-TCPHL-020221-04/2021 | hCoV-19/USA/CA-TCPHL-041921-10/2021 |
| hCoV-19/USA/CA-TCPHL-020221-05/2021 | hCoV-19/USA/CA-TCPHL-041921-11/2021 |
| hCoV-19/USA/CA-TCPHL-020221-10/2021 | hCoV-19/USA/CA-TCPHL-041921-14/2021 |
| hCoV-19/USA/CA-TCPHL-020221-12/2021 | hCoV-19/USA/CA-TCPHL-041921-15/2021 |
| hCoV-19/USA/CA-TCPHL-032921-06/2021 | hCoV-19/USA/CA-TCPHL-041921-16/2021 |
| hCoV-19/USA/CA-TCPHL-032921-07/2021 | hCoV-19/USA/CA-TCPHL-041921-17/2021 |
| hCoV-19/USA/CA-TCPHL-032921-08/2021 | hCoV-19/USA/CA-TCPHL-022221-06/2021 |
| hCoV-19/USA/CA-TCPHL-032921-09/2021 | hCoV-19/USA/CA-TCPHL-022221-07/2021 |
| hCoV-19/USA/CA-TCPHL-032921-10/2021 | hCoV-19/USA/CA-TCPHL-022221-08/2021 |
| hCoV-19/USA/CA-TCPHL-032921-11/2021 | hCoV-19/USA/CA-TCPHL-022221-09/2021 |
| hCoV-19/USA/CA-TCPHL-032921-13/2021 | hCoV-19/USA/CA-TCPHL-022221-10/2021 |
| hCoV-19/USA/CA-TCPHL-032921-14/2021 | hCoV-19/USA/CA-TCPHL-022221-11/2021 |
| hCoV-19/USA/CA-TCPHL-032921-15/2021 | hCoV-19/USA/CA-TCPHL-022221-12/2021 |
| hCoV-19/USA/CA-TCPHL-032921-16/2021 | hCoV-19/USA/CA-TCPHL-022221-13/2021 |
| hCoV-19/USA/CA-TCPHL-032921-17/2021 | hCoV-19/USA/CA-TCPHL-022221-14/2021 |
| hCoV-19/USA/CA-TCPHL-032921-18/2021 | hCoV-19/USA/CA-TCPHL-022221-15/2021 |
| hCoV-19/USA/CA-TCPHL-032921-19/2021 | hCoV-19/USA/CA-TCPHL-031521-04/2021 |
| hCoV-19/USA/CA-TCPHL-032921-20/2021 | hCoV-19/USA/CA-TCPHL-031521-05/2021 |
| hCoV-19/USA/CA-TCPHL-032921-21/2021 | hCoV-19/USA/CA-TCPHL-031521-06/2021 |
| hCoV-19/USA/CA-TCPHL-032921-22/2021 | hCoV-19/USA/CA-TCPHL-031521-07/2021 |
| hCoV-19/USA/CA-TCPHL-032921-23/2021 | hCoV-19/USA/CA-TCPHL-031521-08/2021 |
| hCoV-19/USA/CA-TCPHL-032921-24/2021 | hCoV-19/USA/CA-TCPHL-031521-09/2021 |
| hCoV-19/USA/CA-TCPHL-032921-25/2021 | hCoV-19/USA/CA-TCPHL-031521-10/2021 |
| hCoV-19/USA/CA-TCPHL-032921-26/2021 | hCoV-19/USA/CA-TCPHL-041921-20/2021 |
| hCoV-19/USA/CA-TCPHL-032921-27/2021 | hCoV-19/USA/CA-TCPHL-030121-01/2021 |
| hCoV-19/USA/CA-TCPHL-032921-28/2021 | hCoV-19/USA/CA-TCPHL-030121-02/2021 |
| hCoV-19/USA/CA-TCPHL-032921-29/2021 | hCoV-19/USA/CA-TCPHL-030121-04/2021 |
| hCoV-19/USA/CA-TCPHL-032921-30/2021 | hCoV-19/USA/CA-TCPHL-030121-05/2021 |
| hCoV-19/USA/CA-TCPHL-032921-31/2021 | hCoV-19/USA/CA-TCPHL-030121-06/2021 |
| hCoV-19/USA/CA-TCPHL-040521-07/2021 | hCoV-19/USA/CA-TCPHL-030121-07/2021 |
| hCoV-19/USA/CA-TCPHL-040521-08/2021 | hCoV-19/USA/CA-TCPHL-030121-08/2021 |
| hCoV-19/USA/CA-TCPHL-050321-23/2021 | hCoV-19/USA/CA-TCPHL-030121-09/2021 |
| hCoV-19/USA/CA-TCPHL-050321-24/2021 | hCoV-19/USA/CA-TCPHL-030121-10/2021 |
| hCoV-19/USA/CA-TCPHL-050321-25/2021 | hCoV-19/USA/CA-TCPHL-030121-11/2021 |
| hCoV-19/USA/CA-TCPHL-050321-26/2021 | hCoV-19/USA/CA-TCPHL-030121-12/2021 |
| hCoV-19/USA/CA-TCPHL-050321-27/2021 | hCoV-19/USA/CA-TCPHL-030121-13/2021 |
| hCoV-19/USA/CA-TCPHL-050321-28/2021 | hCoV-19/USA/CA-TCPHL-030121-14/2021 |
| hCoV-19/USA/CA-TCPHL-050321-29/2021 | hCoV-19/USA/CA-TCPHL-030121-15/2021 |
| hCoV-19/USA/CA-TCPHL-050321-31/2021 | hCoV-19/USA/CA-TCPHL-030121-16/2021 |
| hCoV-19/USA/CA-TCPHL-050321-32/2021 | hCoV-19/USA/CA-TCPHL-030121-17/2021 |
| hCoV-19/USA/CA-TCPHL-050321-34/2021 | hCoV-19/USA/CA-TCPHL-030121-18/2021 |
| hCoV-19/USA/CA-TCPHL-050321-35/2021 | hCoV-19/USA/CA-TCPHL-030821-11/2021 |
| hCoV-19/USA/CA-TCPHL-050321-36/2021 | hCoV-19/USA/CA-TCPHL-030821-12/2021 |
| hCoV-19/USA/CA-TCPHL-051021-12/2021 | hCoV-19/USA/CA-TCPHL-030821-13/2021 |
| hCoV-19/USA/CA-TCPHL-051021-14/2021 | hCoV-19/USA/CA-TCPHL-030821-14/2021 |
| hCoV-19/USA/CA-TCPHL-051021-15/2021 | hCoV-19/USA/CA-TCPHL-030821-15/2021 |
| hCoV-19/USA/CA-TCPHL-051021-16/2021 | hCoV-19/USA/CA-TCPHL-030821-16/2021 |
| hCoV-19/USA/CA-TCPHL-051021-17/2021 | hCoV-19/USA/CA-TCPHL-030821-17/2021 |
| hCoV-19/USA/CA-TCPHL-051021-18/2021 | hCoV-19/USA/CA-TCPHL-030821-01/2021 |
| hCoV-19/USA/CA-TCPHL-051021-19/2021 | hCoV-19/USA/CA-TCPHL-030821-02/2021 |
| hCoV-19/USA/CA-TCPHL-051021-20/2021 | hCoV-19/USA/CA-TCPHL-030821-03/2021 |
| hCoV-19/USA/CA-TCPHL-051021-21/2021 | hCoV-19/USA/CA-TCPHL-030821-04/2021 |
| hCoV-19/USA/CA-TCPHL-051021-22/2021 | hCoV-19/USA/CA-TCPHL-030821-05/2021 |
| hCoV-19/USA/CA-TCPHL-051021-23/2021 | hCoV-19/USA/CA-TCPHL-030821-07/2021 |
| hCoV-19/USA/CA-TCPHL-051021-24/2021 | hCoV-19/USA/CA-TCPHL-030821-08/2021 |
| hCoV-19/USA/CA-TCPHL-051021-25/2021 | hCoV-19/USA/CA-TCPHL-030821-09/2021 |
| hCoV-19/USA/CA-TCPHL-051021-26/2021 | hCoV-19/USA/CA-TCPHL-030821-10/2021 |
| hCoV-19/USA/CA-TCPHL-051021-27/2021 | hCoV-19/USA/CA-TCPHL-030821-18/2021 |
| hCoV-19/USA/CA-TCPHL-051021-28/2021 | hCoV-19/USA/CA-TCPHL-030821-19/2021 |
| hCoV-19/USA/CA-TCPHL-051021-29/2021 | hCoV-19/USA/CA-TCPHL-030821-20/2021 |
| hCoV-19/USA/CA-TCPHL-020221-01/2021 | hCoV-19/USA/CA-TCPHL-030821-21/2021 |
| hCoV-19/USA/CA-TCPHL-020221-11/2021 | hCoV-19/USA/CA-TCPHL-031521-01/2021 |
| hCoV-19/USA/CA-TCPHL-032221-17/2021 | hCoV-19/USA/CA-TCPHL-031521-02/2021 |
| hCoV-19/USA/CA-TCPHL-032221-18/2021 | hCoV-19/USA/CA-TCPHL-031521-03/2021 |
| hCoV-19/USA/CA-TCPHL-032221-19/2021 | hCoV-19/USA/CA-TCPHL-032221-09/2021 |
| hCoV-19/USA/CA-TCPHL-032221-20/2021 | hCoV-19/USA/CA-TCPHL-032221-10/2021 |
| hCoV-19/USA/CA-TCPHL-032221-21/2021 | hCoV-19/USA/CA-TCPHL-032221-01/2021 |
| hCoV-19/USA/CA-TCPHL-032221-22/2021 | hCoV-19/USA/CA-TCPHL-032221-02/2021 |
| hCoV-19/USA/CA-TCPHL-032221-23/2021 | hCoV-19/USA/CA-TCPHL-032221-03/2021 |
| hCoV-19/USA/CA-TCPHL-032221-24/2021 | hCoV-19/USA/CA-TCPHL-032221-04/2021 |
| hCoV-19/USA/CA-TCPHL-032221-25/2021 | hCoV-19/USA/CA-TCPHL-032221-05/2021 |
| hCoV-19/USA/CA-TCPHL-032221-30/2021 | hCoV-19/USA/CA-TCPHL-032221-07/2021 |
| hCoV-19/USA/CA-TCPHL-032221-31/2021 | hCoV-19/USA/CA-TCPHL-032221-08/2021 |
| hCoV-19/USA/CA-TCPHL-032921-04/2021 | hCoV-19/USA/CA-TCPHL-041221-21/2021 |
| hCoV-19/USA/CA-TCPHL-032921-05/2021 | hCoV-19/USA/CA-TCPHL-041221-22/2021 |
| hCoV-19/USA/CA-TCPHL-042621-28/2021 | hCoV-19/USA/CA-TCPHL-032921-02/2021 |
| hCoV-19/USA/CA-TCPHL-042621-30/2021 | hCoV-19/USA/CA-TCPHL-032921-03/2021 |
| hCoV-19/USA/CA-TCPHL-042621-31/2021 | hCoV-19/USA/CA-TCPHL-040521-04/2021 |
| hCoV-19/USA/CA-TCPHL-042621-32/2021 | hCoV-19/USA/CA-TCPHL-040521-05/2021 |
| hCoV-19/USA/CA-TCPHL-042621-33/2021 | hCoV-19/USA/CA-TCPHL-040521-01/2021 |
| hCoV-19/USA/CA-TCPHL-042621-34/2021 | hCoV-19/USA/CA-TCPHL-040521-02/2021 |
| hCoV-19/USA/CA-TCPHL-042621-35/2021 | hCoV-19/USA/CA-TCPHL-040521-03/2021 |
| hCoV-19/USA/CA-TCPHL-042621-36/2021 | hCoV-19/USA/CA-TCPHL-040521-06/2021 |
| hCoV-19/USA/CA-TCPHL-042621-37/2021 | hCoV-19/USA/CA-TCPHL-041221-01/2021 |
| hCoV-19/USA/CA-TCPHL-050321-02/2021 | hCoV-19/USA/CA-TCPHL-041221-02/2021 |
| hCoV-19/USA/CA-TCPHL-050321-03/2021 | hCoV-19/USA/CA-TCPHL-041221-03/2021 |
| hCoV-19/USA/CA-TCPHL-050321-04/2021 | hCoV-19/USA/CA-TCPHL-041221-04/2021 |
| hCoV-19/USA/CA-TCPHL-050321-05/2021 | hCoV-19/USA/CA-TCPHL-041921-01/2021 |
| hCoV-19/USA/CA-TCPHL-050321-06/2021 | hCoV-19/USA/CA-TCPHL-041921-02/2021 |
| hCoV-19/USA/CA-TCPHL-050321-07/2021 | hCoV-19/USA/CA-TCPHL-041921-03/2021 |
| hCoV-19/USA/CA-TCPHL-050321-08/2021 | hCoV-19/USA/CA-TCPHL-041921-04/2021 |
| hCoV-19/USA/CA-TCPHL-050321-09/2021 | hCoV-19/USA/CA-TCPHL-041921-05/2021 |
| hCoV-19/USA/CA-TCPHL-050321-10/2021 | hCoV-19/USA/CA-TCPHL-041921-07/2021 |
| hCoV-19/USA/CA-TCPHL-050321-11/2021 | hCoV-19/USA/CA-TCPHL-041921-08/2021 |
| hCoV-19/USA/CA-TCPHL-050321-12/2021 | hCoV-19/USA/CA-TCPHL-041921-35/2021 |
| hCoV-19/USA/CA-TCPHL-050321-14/2021 | hCoV-19/USA/CA-TCPHL-042621-04/2021 |
| hCoV-19/USA/CA-TCPHL-050321-15/2021 | hCoV-19/USA/CA-TCPHL-042621-01/2021 |
| hCoV-19/USA/CA-TCPHL-050321-16/2021 | hCoV-19/USA/CA-TCPHL-042621-02/2021 |
| hCoV-19/USA/CA-TCPHL-050321-17/2021 | hCoV-19/USA/CA-TCPHL-042621-03/2021 |
| hCoV-19/USA/CA-TCPHL-050321-18/2021 | hCoV-19/USA/CA-TCPHL-042621-05/2021 |
| hCoV-19/USA/CA-TCPHL-050321-19/2021 | hCoV-19/USA/CA-TCPHL-042621-06/2021 |
| hCoV-19/USA/CA-TCPHL-050321-20/2021 | hCoV-19/USA/CA-TCPHL-042621-07/2021 |
| hCoV-19/USA/CA-TCPHL-050321-21/2021 | hCoV-19/USA/CA-TCPHL-051021-09/2021 |
| hCoV-19/USA/CA-TCPHL-050321-22/2021 | hCoV-19/USA/CA-TCPHL-042621-08/2021 |
| hCoV-19/USA/CA-TCPHL-031521-16/2021 | hCoV-19/USA/CA-TCPHL-050321-01/2021 |
| hCoV-19/USA/CA-TCPHL-031521-18/2021 | hCoV-19/USA/CA-TCPHL-051021-04/2021 |
| hCoV-19/USA/CA-TCPHL-031521-19/2021 | hCoV-19/USA/CA-TCPHL-051021-01/2021 |
| hCoV-19/USA/CA-TCPHL-031521-20/2021 | hCoV-19/USA/CA-TCPHL-051021-03/2021 |
| hCoV-19/USA/CA-TCPHL-031521-21/2021 | hCoV-19/USA/CA-TCPHL-051021-06/2021 |
| hCoV-19/USA/CA-TCPHL-031521-22/2021 | hCoV-19/USA/CA-TCPHL-051021-08/2021 |
| hCoV-19/USA/CA-TCPHL-032221-11/2021 | hCoV-19/USA/CA-TCPHL-032921-12/2021 |
| hCoV-19/USA/CA-TCPHL-050321-30/2021 | hCoV-19/USA/CA-TCPHL-041921-28/2021 |
| hCoV-19/USA/CA-TCPHL-041221-15/2021 | hCoV-19/USA/CA-TCPHL-032921-19/2021 |
| hCoV-19/USA/CA-TCPHL-050321-26/2021 | hCoV-19/USA/CA-TCPHL-050321-28/2021 |
| hCoV-19/USA/CA-TCPHL-050321-31/2021 | hCoV-19/USA/CA-TCPHL-051021-18/2021 |
| hCoV-19/USA/CA-TCPHL-051021-20/2021 | hCoV-19/USA/CA-TCPHL-030121-13/2021 |
| hCoV-19/USA/CA-TCPHL-030121-14/2021 | hCoV-19/USA/CA-TCPHL-030121-15/2021 |
| hCoV-19/USA/CA-TCPHL-040521-01/2021 | hCoV-19/USA/CA-TCPHL-040521-02/2021 |
| hCoV-19/USA/CA-TCPHL-031521-15/2021 | hCoV-19/USA/CA-TCPHL-020221-08/2021 |
